# Informed Consent for Phase I Oncology Trials: Form, Substance and Signature

**DOI:** 10.14740/jocmr1803w

**Published:** 2014-03-31

**Authors:** Laeeq Malik, Alex Mejia

**Affiliations:** a Institute for Drug Development, Cancer Therapy and Research Center (CTRC), University of Texas Health Science Center, San Antonio, TX, USA

**Keywords:** Informed consent, Patient, Phase I, Oncology, Trial

## Abstract

**Background:**

Federal regulations state consent information should be understandable to participants; concerns have been voiced about the quality of informed consent forms (ICFs) in oncology trials.

**Methods:**

The content of ICFs for phase I studies that were conducted at a tertiary care cancer center over 3 years’ period was reviewed. Information pertaining to the length of the ICF, description of study purpose, research regimen/methods, treatment agent, potential risks, benefits and alternatives to the research was extracted.

**Results:**

In total, 54 ICFs for phase I trials approved by the local Institutional Review Board were reviewed. Median length of ICF was 20 pages. Nearly one half of the forms (57.4%) were of first-in-human phase I studies. The main goal of research was explicitly stated as safety testing in 59.2% forms, while 37.1% studies described primary objective as dose finding. All of the forms identified serious risks, unexpected risks, possibility of death and risks to pregnant and or lactating women. A detailed estimation of the frequency or intensity of risks (range 3-8 pages) was provided qualitatively or quantitatively if known. Information regarding mechanism of action of investigational agent, study schema, dose escalation, loss of time/energy and possibility of receiving sub-therapeutic dose was missing in significant number of forms.

**Conclusion:**

We found that these ICFs were compliant with approved guidelines and provided a thorough description of risks or potential benefits. However, there still remains room for improvement, so patients can make better informed decisions.

## Introduction

Written informed consent is an essential element and a requirement in the conduct of clinical research [1]. To obtain an informed consent, potential study participants should be provided with an Institutional Review Board approved consent form to review, so that the subject can make an informed judgment about participation. The informed consent forms (ICFs) are written in accordance with Declaration of Helsinki and International Conference on Harmonization (ICH) guidelines for Good Clinical Practice and in this country, comply with Title 21 of the Code of Federal Regulation [2]. For many years, ethical concerns have been voiced by several groups about the quality of ICFs in oncology trials. While some studies have shown that the length of the ICFs has increased significantly, other critics have focused on readability, content difficulty and comprehension [3-5]. Patients with advanced cancer often have decreased comprehension, memory change and concentration difficulty, which could be related to prior treatment [6].

In view of scientific objectives, the likelihood of direct benefit from classical phase I trials is considered to be low [7, 8]. Unfortunately unrealistic optimism, therapeutic misconception and misestimation are common among cancer patients with advanced disease [9-14]. It is unclear if some of these misunderstandings stem from the ICF [15]. In order to overcome these challenges, various strategies have been proposed, including simplifying the ICF [16, 17]. It is critically important to determine whether the gaps in study participants’ understanding are related to ICFs.

## Methods

We reviewed ICFs of 54 consecutive phase I studies that were conducted between 2011 and 2013 at a tertiary care cancer center. Information pertaining to the length of the document, description of study purpose, research regimen/methods, treatment agent, potential risks, benefits and alternatives to the research was extracted. We excluded pediatric trials, non-therapeutic trials (namely, biomarker or observational studies), those without electronically accessible ICFs and radiation therapy studies. Data analysis was performed using Microsoft Excel 2003.

## Results

The median length of ICF was 20 pages (range 14-27). Nearly one half of the forms (57.4%) were of first-in-human phase I studies, whilst almost one-quarter (22.2%) of the forms involved Food and Drug Administration approved agents. All of the ICFs clearly stated the voluntary nature of participation, and made a distinction between standard of care and research procedures. The main goal of research was explicitly stated as safety testing in 59.2% forms, while 37.1% studies described primary objective as dose finding. Most of the forms (74%) stated clearly that individual participants may not benefit, only 26% studies (including both approved and non-approved agents) worded that their disease condition may improve. Potential benefit to society through generalizable knowledge was mentioned in all the forms. All of the forms identified serious risks, unexpected risks, possibility of death and risks to pregnant and/or lactating women. A detailed estimation of the frequency or intensity of risks (range 3-8 pages) was provided qualitatively or quantitatively in 77.7% forms, whereas the remaining 22.2% described the frequency of risks as unknown. A clearly prominent statement about compensation to subjects and alternative treatment options was provided in all of the forms. In 74.4% studies, the sponsor made a commitment to cover the cost of any research related injury; however, 25.6% studies (mainly investigator initiated and co-operative group) required patients to be responsible for these costs.

## Discussion

The results of our review suggest some important areas for improvement in consent forms for phase I oncology trials. It was noticed that the mechanism of action of the investigational agent was only provided in 22.2% forms. Subjects have a right to written information about the drug’s known properties, which is an important part of informed consent process and therapeutic education. Study schema and treatment calendar were provided only in 25% ICFs in our analysis. Complex study designs and dose escalations were explained over several pages in ICFs which would likely extend beyond the comprehension of sick cancer patients. When compounded by limited literacy, complicated explanations of study design may become a major source of miscommunication between patients and researchers. Inclusion of simplified study and dose escalation schemas could aid patients in understanding the trial schedule, procedures and timelines. Visual communication and not just verbal is important to provide better understanding to potential study participants, many of whom have limited health literacy.

In order to improve participant’s comprehension, in addition to being written in lay terminology, consent forms could be kept brief and direct. The length of consent document remains an important concern, as it may pose a challenge for cancer patients to find out important necessary information and may well exceed patient’s memory capacity. Although there seems to be consensus on what type of information patients would prefer to have in order to make an informed decision about participation in research, the level of details they would prefer is unknown [18]. In a recently completed randomized controlled study, a short consent form (only five pages) compared favorably with the standard form in all outcome measures, including patients’ understanding, levels of recall, concerns, trust and voluntariness [19]. Involvement of a patient advocate in the development of ICFs may further help in simplifying the forms and selection of information that patients find necessary to know. The National Cancer Institute (NCI) has developed recommendations and a template to simplify ICF and enhance the research participant’s understanding of the consent form [20]. The NCI strongly recommends that consent forms should not exceed six to nine pages. ICFs can be made more concise by including in the main consent document all the essential elements (Table 1) that makes the study different from standard of care. Detailed information on standard of care procedures, tables, lists, appendices, and so on can be provided as a supplemental form or handbook. The amount of supplemental information that individual subjects may want will vary, but should always be available to them. Unnecessary repetition of information and use of professional jargon, acronyms and abbreviations should be avoided.

**Table 1:** Federally Required Elements of Informed Consent [21]

1.	A statement that the study involves research, an explanation of the purposes of the research and the expected duration of the subject’s participation, a description of the procedures to be followed and identification of any procedures which are experimental;
2.	A description of any reasonably foreseeable risks or discomforts to the subject;
3.	A description of any benefits to the subject or to others which may reasonably be expected from the research;
4.	A disclosure of appropriate alternative procedures or courses of treatment, if any, that might be advantageous to the subject;
5.	A statement describing the extent, if any, to which confidentiality of records identifying the subject will be maintained;
6.	For research involving more than minimal risk, an explanation as to whether any compensation and an explanation as to whether any medical treatments are available if injury occurs and, if so, what they consist of, or where further information may be obtained;
7.	An explanation of whom to contact for answers to pertinent questions about the research and research subjects’ rights, and whom to contact in the event of a research-related injury to the subject; and
8.	A statement that participation is voluntary, refusal to participate will involve no penalty or loss of benefits to which the subject is otherwise entitled and the subject may discontinue participation at any time without penalty or loss of benefits to which the subject is otherwise entitled.

We also noticed that a limited number of consent forms (40.7%) reported nonphysical effects from participating in phase I studies such as loss of time at work/home or the possibility of being asked sensitive/private questions. Early phase clinical trials are very time-consuming for patients with numerous clinic visits for the purpose of performing pharmacokinetic/pharmacodynamics studies, laboratory tests, safety checks and treatment. As time is the most valuable commodity for the cancer patients, consent forms must clearly state frequency and expected duration of all clinic visits.

In summary, our review of ICFs for 54 phase I studies from a single institution shows that they contained the content as recommended by ICH/GCP guidelines and federal law [21], but not all contained information that would be beneficial for patient participation. They provided a thorough description of potential risks and did not overpromise the benefit. Further efforts are needed to incorporate the missing information in a supplementary form (Table 2) and reduce the main document length which will help patients to make better informed decisions.

**Table 2:** Potentially Helpful Information Missing From ICFs of Phase I Studies

Type of information	Missing in this review (%)
Mechanism of action of investigational agent	77.8
Study scheme and design	75.0
Loss of time/energy	59.3
Dose escalation and possibility of receiving sub- therapeutic dose	37.0
Expected duration of each visit	25.0
